# Notice by the Committee of Arrangements

**Published:** 1878-05

**Authors:** 


**The Chairmen and Secretaries of the several Sections of the American Medical Association, are requested to send in (by title) the papers to come before them, with the time required for reading, also all gentlemen having papers to present, not referable to the Sections, will send as above, without delay, to**

**THOS. F. ROCHESTER,**
**Ch. of Com. of Arrangements, Buffalo, N. Y**

**Medical Journals are requested to copy, and give prominence to this notice.**

